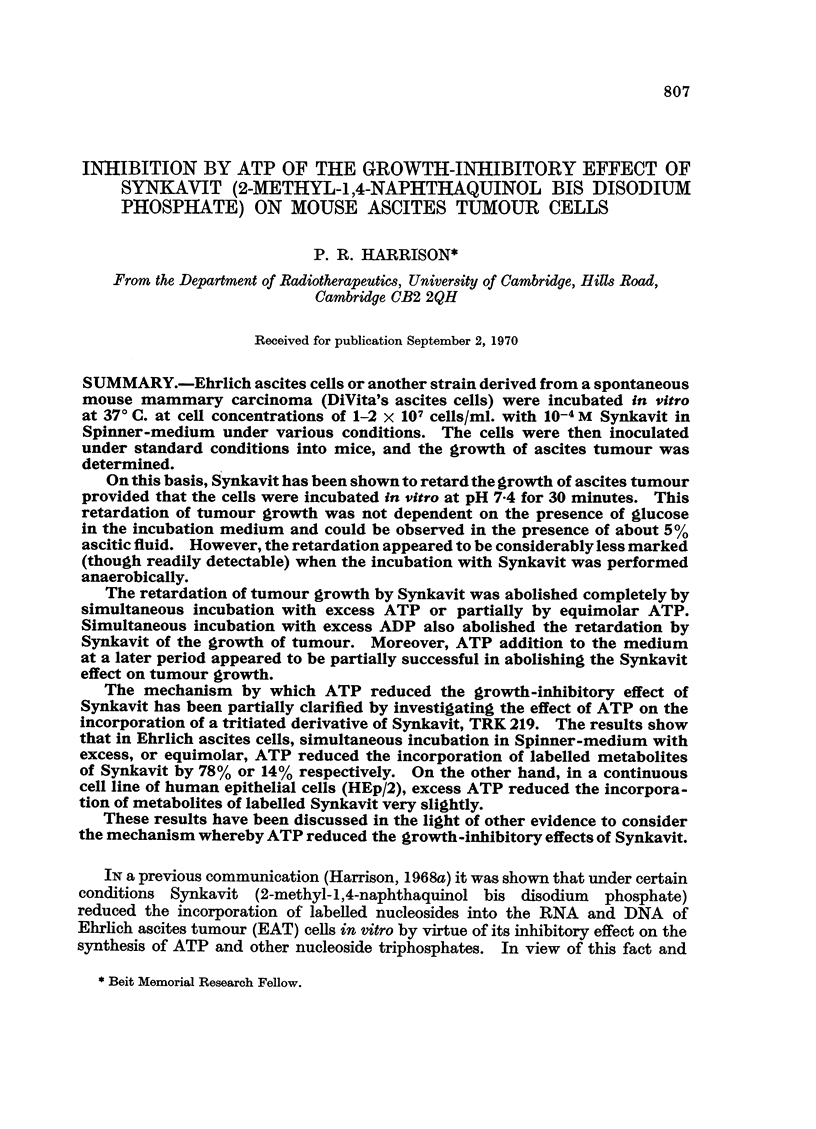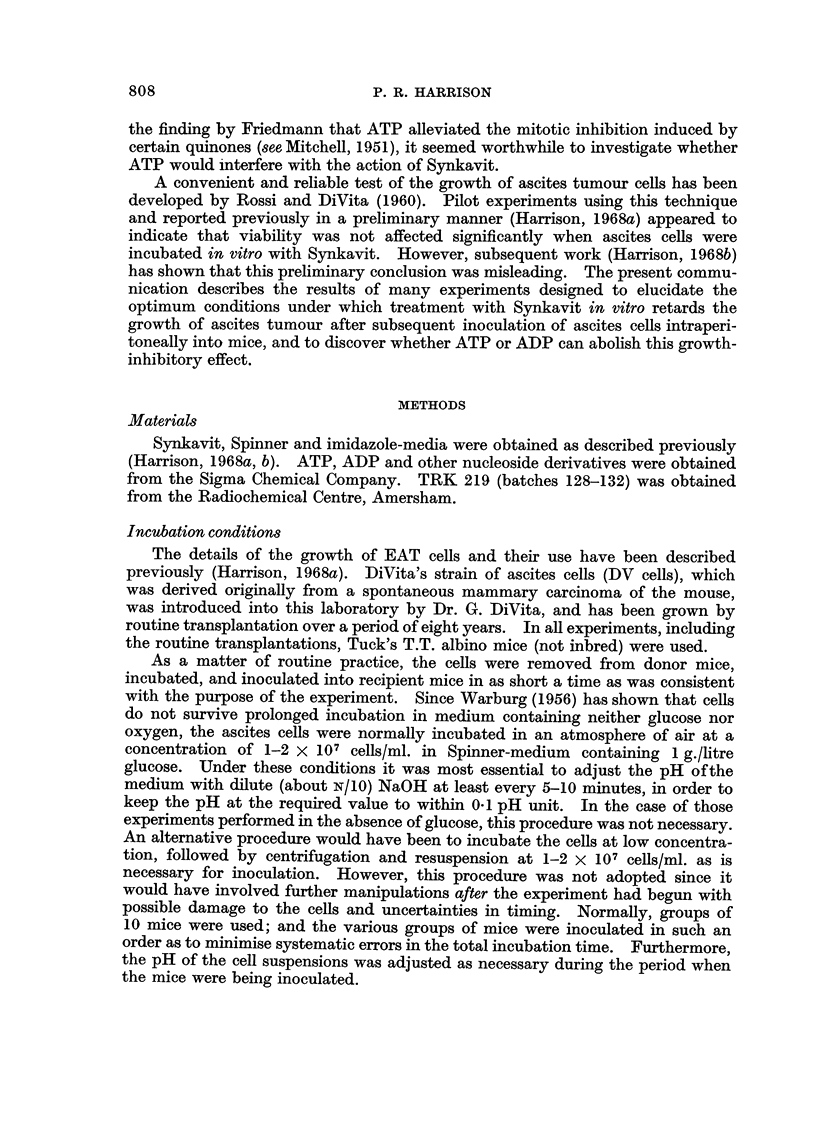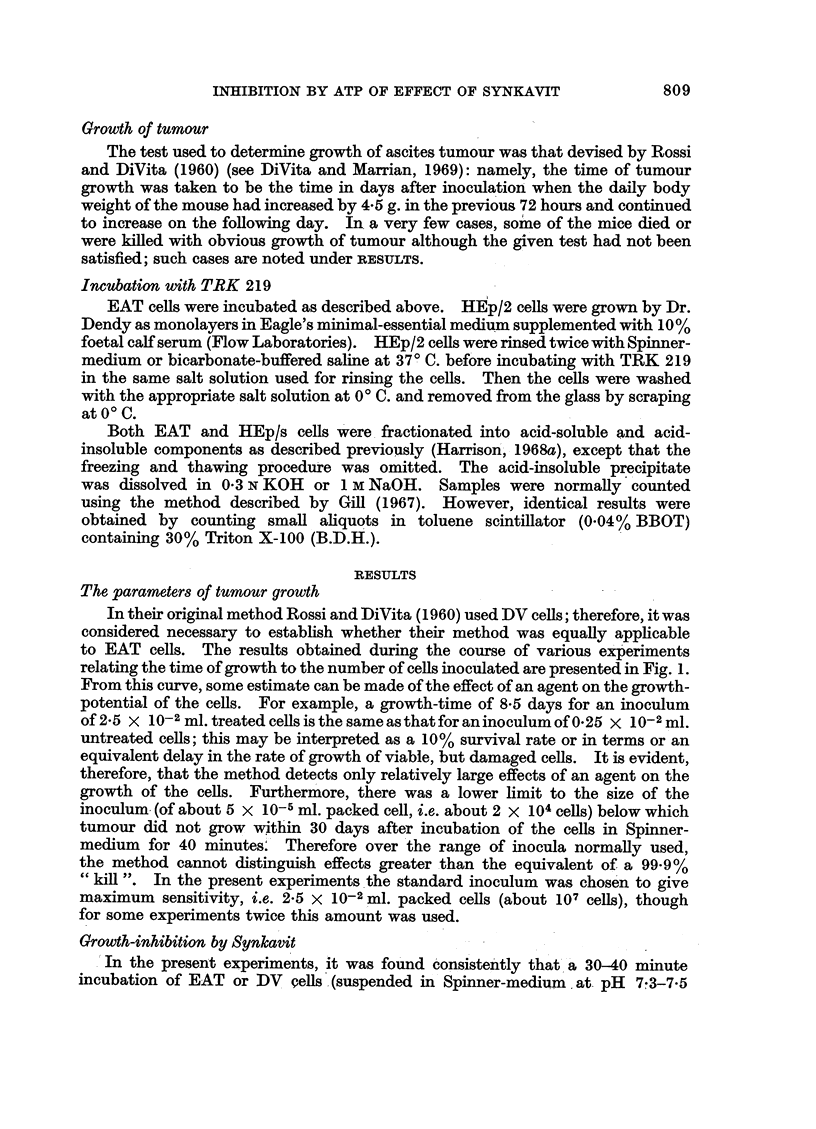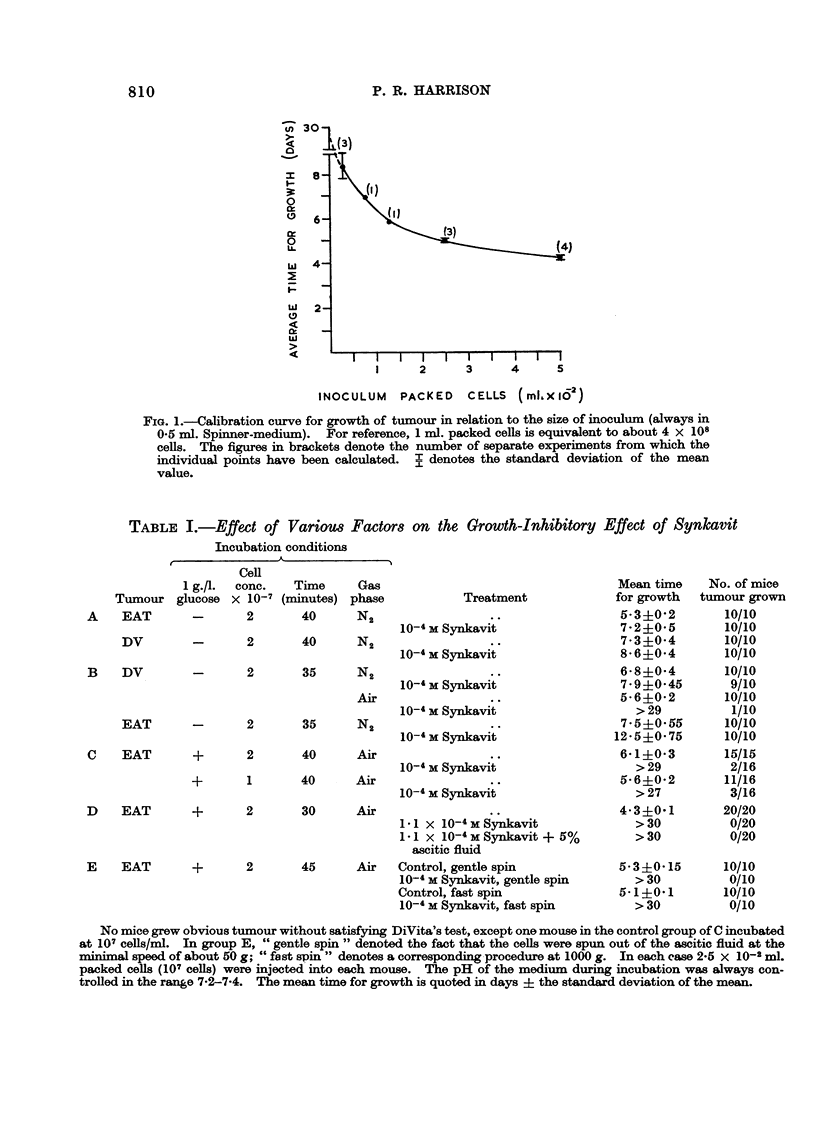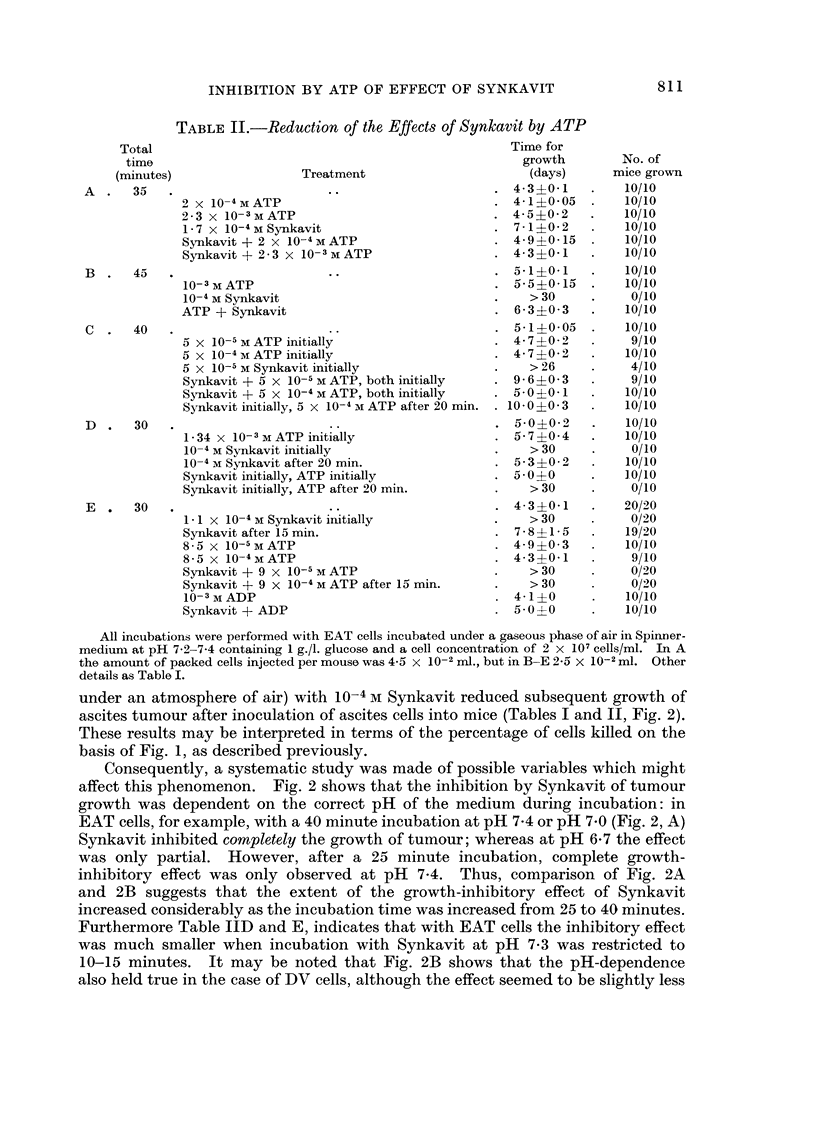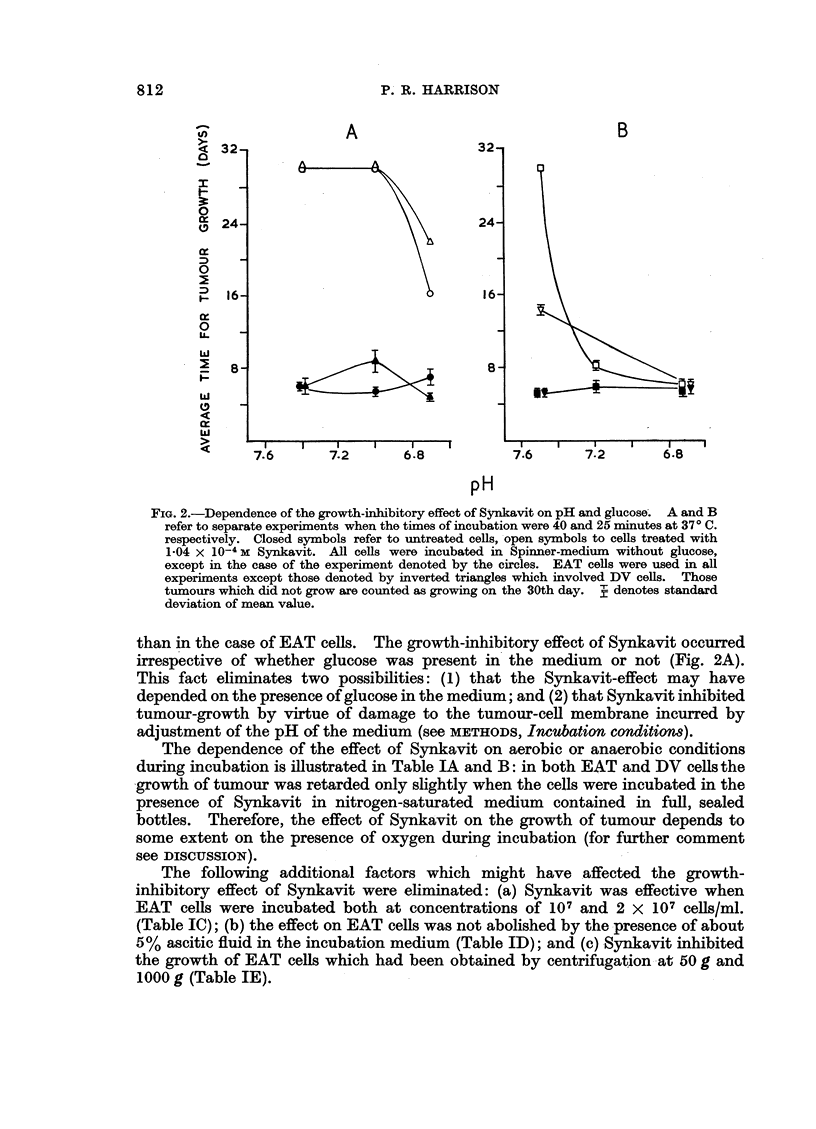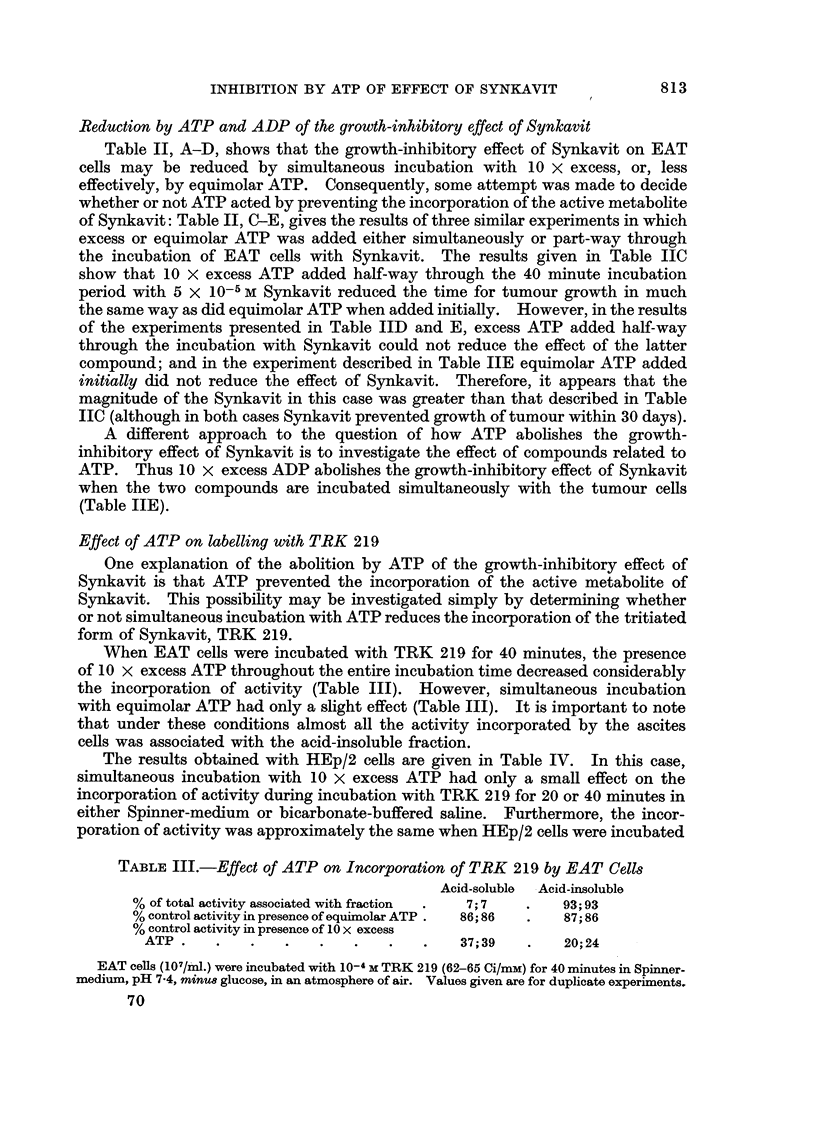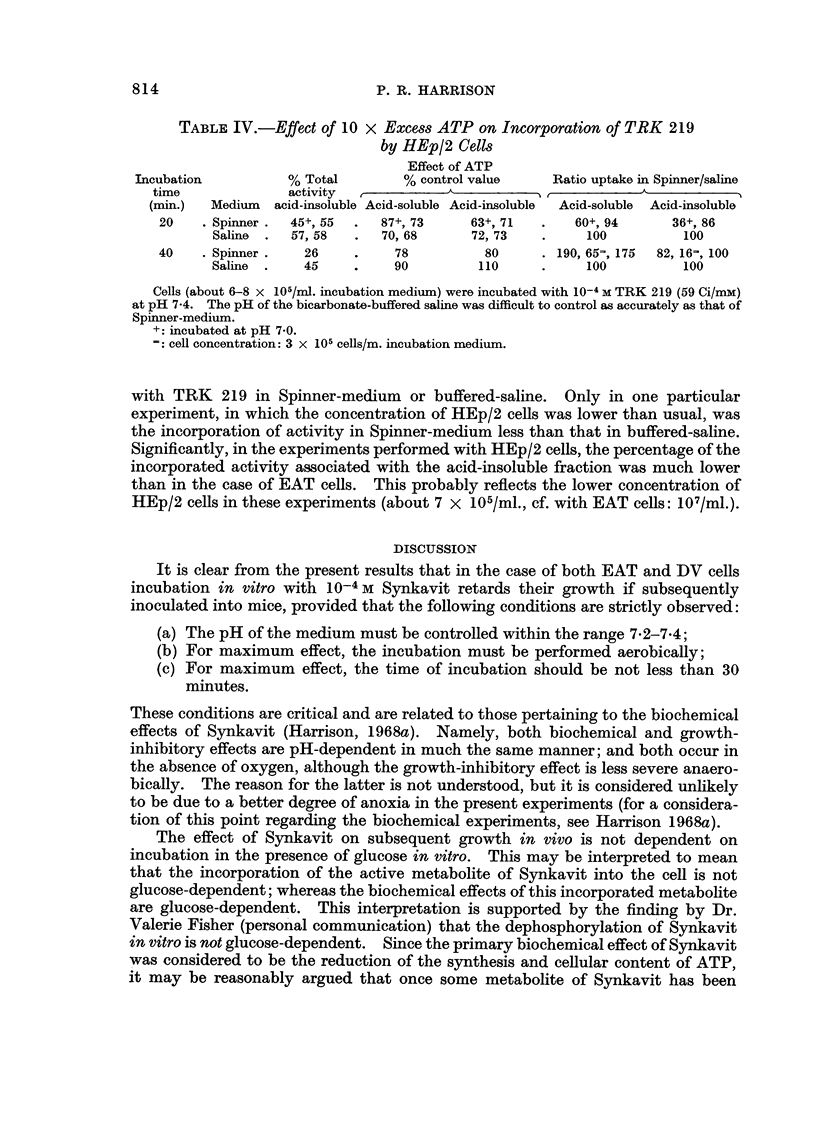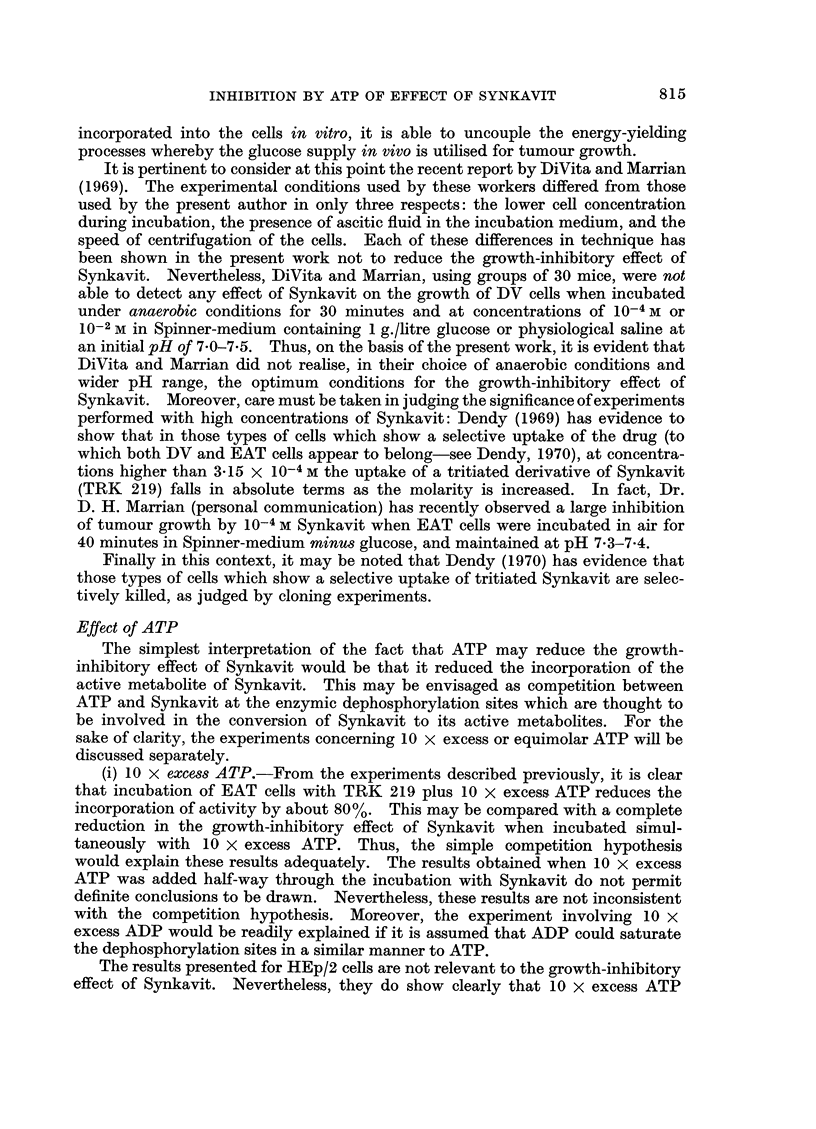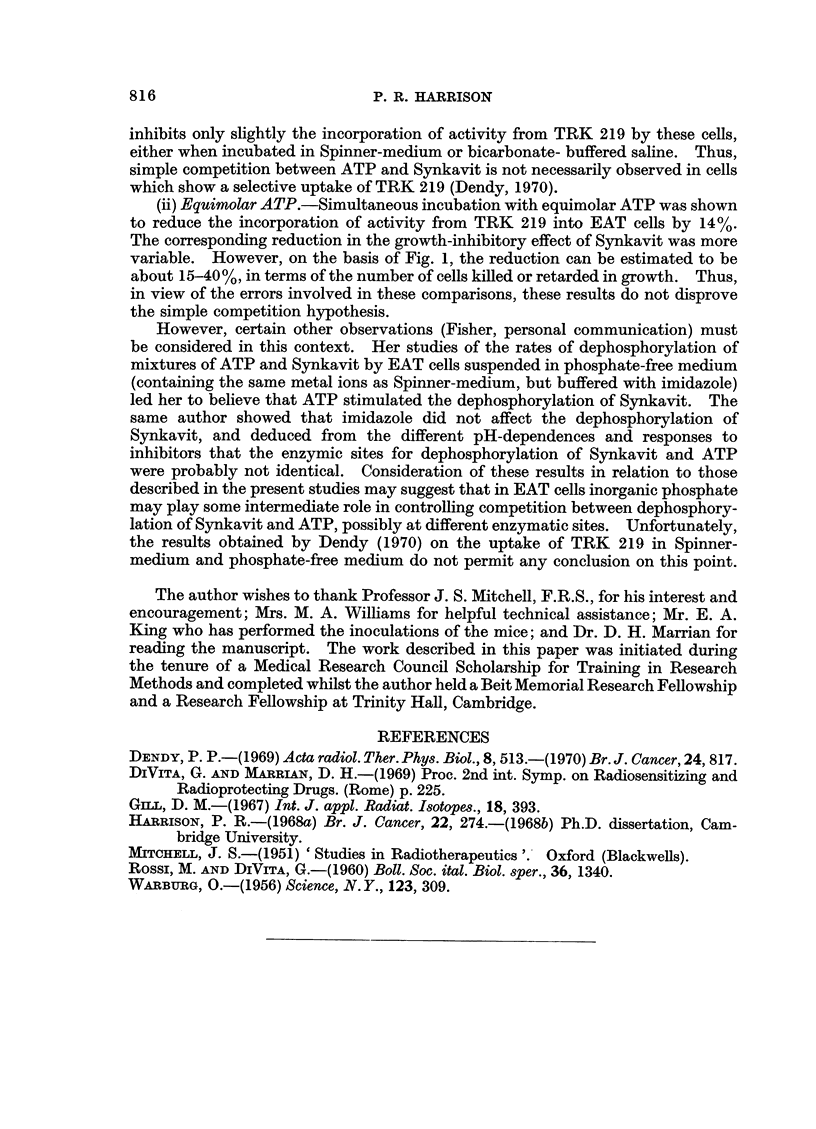# Inhibition ATP of the Growth-inhibitory Effect of Synkavit (2-methyl-1,4-naphthaquinol Bis Disodium Phosphate) on Mouse Ascites Tumour Cells

**Published:** 1970-12

**Authors:** P. R. Harrison

## Abstract

Ehrlich ascites cells or another strain derived from a spontaneous mouse mammary carcinoma (DiVita's ascites cells) were incubated *in vitro* at 37° C. at cell concentrations of 1-2 × 10^7^ cells/ml. with 10^-4^M Synkavit in Spinner-medium under various conditions. The cells were then inoculated under standard conditions into mice, and the growth of ascites tumour was determined.

On this basis, Synkavit has been shown to retard the growth of ascites tumour provided that the cells were incubated in *vitro* at pH 7.4 for 30 minutes. This retardation of tumour growth was not dependent on the presence of glucose in the incubation medium and could be observed in the presence of about 5% ascitic fluid. However, the retardation appeared to be considerably less marked (though readily detectable) when the incubation with Synkavit was performed anaerobically.

The retardation of tumour growth by Synkavit was abolished completely by simultaneous incubation with excess ATP or partially by equimolar ATP. Simultaneous incubation with excess ADP also abolished the retardation by Synkavit of the growth of tumour. Moreover, ATP addition to the medium at a later period appeared to be partially successful in abolishing the Synkavit effect on tumour growth.

The mechanism by which ATP reduced the growth-inhibitory effect of Synkavit has been partially clarified by investigating the effect of ATP on the incorporation of a tritiated derivative of Synkavit, TRK 219. The results show that in Ehrlich ascites cells, simultaneous incubation in Spinner-medium with excess, or equimolar, ATP reduced the incorporation of labelled metabolites of Synkavit by 78% or 14% respectively. On the other hand, in a continuous cell line of human epithelial cells (HEp/2), excess ATP reduced the incorporation of metabolites of labelled Synkavit very slightly.

These results have been discussed in the light of other evidence to consider the mechanism whereby ATP reduced the growth-inhibitory effects of Synkavit.


### INHIBITION BY ATP OF THE GROWTH-INHIBITORY EFFECT OF SYNKAVIT (2-METHYL-1,4-NAPHTHAQUINOL BIS DISODIUM PHOSPHATE) ON MOUSE ASCITES TUMOUR CELLS

P. R. HARRISON*

*From the Department of Radiotherapeutics, University of Cambridge, Hills Road, Cambridge CB2 2QH*

Received for publication September 2, 1970

**SUMMARY.—Ehrlich ascites cells or another strain derived from a spontaneous mouse mammary carcinoma (DiVita's ascites cells) were incubated *in vitro* at 37° C. at cell concentrations of 1–2 × $10^7$ cells/ml. with $10^{-4}$ M Synkavit in Spinner-medium under various conditions. The cells were then inoculated under standard conditions into mice, and the growth of ascites tumour was determined.**

**On this basis, Synkavit has been shown to retard the growth of ascites tumour provided that the cells were incubated *in vitro* at pH 7·4 for 30 minutes. This retardation of tumour growth was not dependent on the presence of glucose in the incubation medium and could be observed in the presence of about 5% ascitic fluid. However, the retardation appeared to be considerably less marked (though readily detectable) when the incubation with Synkavit was performed anaerobically.**

**The retardation of tumour growth by Synkavit was abolished completely by simultaneous incubation with excess ATP or partially by equimolar ATP. Simultaneous incubation with excess ADP also abolished the retardation by Synkavit of the growth of tumour. Moreover, ATP addition to the medium at a later period appeared to be partially successful in abolishing the Synkavit effect on tumour growth.**

**The mechanism by which ATP reduced the growth-inhibitory effect of Synkavit has been partially clarified by investigating the effect of ATP on the incorporation of a tritiated derivative of Synkavit, TRK 219. The results show that in Ehrlich ascites cells, simultaneous incubation in Spinner-medium with excess, or equimolar, ATP reduced the incorporation of labelled metabolites of Synkavit by 78% or 14% respectively. On the other hand, in a continuous cell line of human epithelial cells (HEp/2), excess ATP reduced the incorporation of metabolites of labelled Synkavit very slightly.**

**These results have been discussed in the light of other evidence to consider the mechanism whereby ATP reduced the growth-inhibitory effects of Synkavit.**

IN a previous communication (Harrison, 1968*a*) it was shown that under certain conditions Synkavit (2-methyl-1,4-naphthaquinol bis disodium phosphate) reduced the incorporation of labelled nucleosides into the RNA and DNA of Ehrlich ascites tumour (EAT) cells *in vitro* by virtue of its inhibitory effect on the synthesis of ATP and other nucleoside triphosphates. In view of this fact and

* Beit Memorial Research Fellow.

the finding by Friedmann that ATP alleviated the mitotic inhibition induced by certain quinones (*see* Mitchell, 1951), it seemed worthwhile to investigate whether ATP would interfere with the action of Synkavit.

A convenient and reliable test of the growth of ascites tumour cells has been developed by Rossi and DiVita (1960). Pilot experiments using this technique and reported previously in a preliminary manner (Harrison, 1968*a*) appeared to indicate that viability was not affected significantly when ascites cells were incubated *in vitro* with Synkavit. However, subsequent work (Harrison, 1968*b*) has shown that this preliminary conclusion was misleading. The present communication describes the results of many experiments designed to elucidate the optimum conditions under which treatment with Synkavit *in vitro* retards the growth of ascites tumour after subsequent inoculation of ascites cells intraperitoneally into mice, and to discover whether ATP or ADP can abolish this growth-inhibitory effect.

### METHODS

### *Materials*

Synkavit, Spinner and imidazole-media were obtained as described previously (Harrison, 1968*a*, *b*). ATP, ADP and other nucleoside derivatives were obtained from the Sigma Chemical Company. TRK 219 (batches 128–132) was obtained from the Radiochemical Centre, Amersham.

### *Incubation conditions*

The details of the growth of EAT cells and their use have been described previously (Harrison, 1968*a*). DiVita's strain of ascites cells (DV cells), which was derived originally from a spontaneous mammary carcinoma of the mouse, was introduced into this laboratory by Dr. G. DiVita, and has been grown by routine transplantation over a period of eight years. In all experiments, including the routine transplantations, Tuck's T.T. albino mice (not inbred) were used.

As a matter of routine practice, the cells were removed from donor mice, incubated, and inoculated into recipient mice in as short a time as was consistent with the purpose of the experiment. Since Warburg (1956) has shown that cells do not survive prolonged incubation in medium containing neither glucose nor oxygen, the ascites cells were normally incubated in an atmosphere of air at a concentration of $1–2 \times 10^7$ cells/ml. in Spinner-medium containing 1 g./litre glucose. Under these conditions it was most essential to adjust the pH of the medium with dilute (about N/10) NaOH at least every 5–10 minutes, in order to keep the pH at the required value to within 0·1 pH unit. In the case of those experiments performed in the absence of glucose, this procedure was not necessary. An alternative procedure would have been to incubate the cells at low concentration, followed by centrifugation and resuspension at $1–2 \times 10^7$ cells/ml. as is necessary for inoculation. However, this procedure was not adopted since it would have involved further manipulations *after* the experiment had begun with possible damage to the cells and uncertainties in timing. Normally, groups of 10 mice were used; and the various groups of mice were inoculated in such an order as to minimise systematic errors in the total incubation time. Furthermore, the pH of the cell suspensions was adjusted as necessary during the period when the mice were being inoculated.

*Growth of tumour*

The test used to determine growth of ascites tumour was that devised by Rossi and DiVita (1960) (see DiVita and Marrian, 1969): namely, the time of tumour growth was taken to be the time in days after inoculation when the daily body weight of the mouse had increased by 4·5 g. in the previous 72 hours and continued to increase on the following day. In a very few cases, some of the mice died or were killed with obvious growth of tumour although the given test had not been satisfied; such cases are noted under RESULTS.

*Incubation with TRK 219*

EAT cells were incubated as described above. HEp/2 cells were grown by Dr. Dendy as monolayers in Eagle's minimal-essential medium supplemented with 10% foetal calf serum (Flow Laboratories). HEp/2 cells were rinsed twice with Spinner-medium or bicarbonate-buffered saline at 37° C. before incubating with TRK 219 in the same salt solution used for rinsing the cells. Then the cells were washed with the appropriate salt solution at 0° C. and removed from the glass by scraping at 0° C.

Both EAT and HEp/s cells were fractionated into acid-soluble and acid-insoluble components as described previously (Harrison, 1968*a*), except that the freezing and thawing procedure was omitted. The acid-insoluble precipitate was dissolved in 0·3 N KOH or 1 M NaOH. Samples were normally counted using the method described by Gill (1967). However, identical results were obtained by counting small aliquots in toluene scintillator (0·04% BBOT) containing 30% Triton X-100 (B.D.H.).

### RESULTS

*The parameters of tumour growth*

In their original method Rossi and DiVita (1960) used DV cells; therefore, it was considered necessary to establish whether their method was equally applicable to EAT cells. The results obtained during the course of various experiments relating the time of growth to the number of cells inoculated are presented in Fig. 1. From this curve, some estimate can be made of the effect of an agent on the growth-potential of the cells. For example, a growth-time of 8·5 days for an inoculum of $2{\cdot}5 \times 10^{-2}$ ml. treated cells is the same as that for an inoculum of $0{\cdot}25 \times 10^{-2}$ ml. untreated cells; this may be interpreted as a 10% survival rate or in terms or an equivalent delay in the rate of growth of viable, but damaged cells. It is evident, therefore, that the method detects only relatively large effects of an agent on the growth of the cells. Furthermore, there was a lower limit to the size of the inoculum (of about $5 \times 10^{-5}$ ml. packed cell, *i.e.* about $2 \times 10^{4}$ cells) below which tumour did not grow within 30 days after incubation of the cells in Spinner-medium for 40 minutes. Therefore over the range of inocula normally used, the method cannot distinguish effects greater than the equivalent of a 99·9% "kill". In the present experiments the standard inoculum was chosen to give maximum sensitivity, *i.e.* $2{\cdot}5 \times 10^{-2}$ ml. packed cells (about $10^{7}$ cells), though for some experiments twice this amount was used.

*Growth-inhibition by Synkavit*

In the present experiments, it was found consistently that a 30–40 minute incubation of EAT or DV cells (suspended in Spinner-medium at pH 7·3–7·5

FIG. 1.—Calibration curve for growth of tumour in relation to the size of inoculum (always in 0·5 ml. Spinner-medium). For reference, 1 ml. packed cells is equivalent to about 4 × $10^8$ cells. The figures in brackets denote the number of separate experiments from which the individual points have been calculated. ⌶ denotes the standard deviation of the mean value.

TABLE I.—*Effect of Various Factors on the Growth-Inhibitory Effect of Synkavit*

| | | Incubation conditions | | | | | | |
|---|---|---|---|---|---|---|---|---|
| | Tumour | 1 g./l. glucose | Cell conc. × $10^{-7}$ | Time (minutes) | Gas phase | Treatment | Mean time for growth | No. of mice tumour grown |
| A | EAT | — | 2 | 40 | $N_2$ | .. | 5·3±0·2 | 10/10 |
| | | | | | | $10^{-4}$ M Synkavit | 7·2±0·5 | 10/10 |
| | DV | — | 2 | 40 | $N_2$ | .. | 7·3±0·4 | 10/10 |
| | | | | | | $10^{-4}$ M Synkavit | 8·6±0·4 | 10/10 |
| B | DV | — | 2 | 35 | $N_2$ | .. | 6·8±0·4 | 10/10 |
| | | | | | | $10^{-4}$ M Synkavit | 7·9±0·45 | 9/10 |
| | | | | | Air | .. | 5·6±0·2 | 10/10 |
| | | | | | | $10^{-4}$ M Synkavit | >29 | 1/10 |
| | EAT | — | 2 | 35 | $N_2$ | .. | 7·5±0·55 | 10/10 |
| | | | | | | $10^{-4}$ M Synkavit | 12·5±0·75 | 10/10 |
| C | EAT | + | 2 | 40 | Air | .. | 6·1±0·3 | 15/15 |
| | | | | | | $10^{-4}$ M Synkavit | >29 | 2/16 |
| | | + | 1 | 40 | Air | .. | 5·6±0·2 | 11/16 |
| | | | | | | $10^{-4}$ M Synkavit | >27 | 3/16 |
| D | EAT | + | 2 | 30 | Air | .. | 4·3±0·1 | 20/20 |
| | | | | | | 1·1 × $10^{-4}$ M Synkavit | >30 | 0/20 |
| | | | | | | 1·1 × $10^{-4}$ M Synkavit + 5% ascitic fluid | >30 | 0/20 |
| E | EAT | + | 2 | 45 | Air | Control, gentle spin | 5·3±0·15 | 10/10 |
| | | | | | | $10^{-4}$ M Synkavit, gentle spin | >30 | 0/10 |
| | | | | | | Control, fast spin | 5·1±0·1 | 10/10 |
| | | | | | | $10^{-4}$ M Synkavit, fast spin | >30 | 0/10 |

No mice grew obvious tumour without satisfying DiVita's test, except one mouse in the control group of C incubated at $10^7$ cells/ml. In group E, " gentle spin " denoted the fact that the cells were spun out of the ascitic fluid at the minimal speed of about 50 g; " fast spin " denotes a corresponding procedure at 1000 g. In each case 2·5 × $10^{-2}$ ml. packed cells ($10^7$ cells) were injected into each mouse. The pH of the medium during incubation was always controlled in the range 7·2–7·4. The mean time for growth is quoted in days ± the standard deviation of the mean.

TABLE II.—*Reduction of the Effects of Synkavit by ATP*

| | Total time (minutes) | Treatment | Time for growth (days) | No. of mice grown |
|---|---|---|---|---|
| A | 35 | .. | 4·3±0·1 | 10/10 |
| | | $2 \times 10^{-4}$ M ATP | 4·1±0·05 | 10/10 |
| | | $2·3 \times 10^{-3}$ M ATP | 4·5±0·2 | 10/10 |
| | | $1·7 \times 10^{-4}$ M Synkavit | 7·1±0·2 | 10/10 |
| | | Synkavit + $2 \times 10^{-4}$ M ATP | 4·9±0·15 | 10/10 |
| | | Synkavit + $2·3 \times 10^{-3}$ M ATP | 4·3±0·1 | 10/10 |
| B | 45 | .. | 5·1±0·1 | 10/10 |
| | | $10^{-3}$ M ATP | 5·5±0·15 | 10/10 |
| | | $10^{-4}$ M Synkavit | >30 | 0/10 |
| | | ATP + Synkavit | 6·3±0·3 | 10/10 |
| C | 40 | .. | 5·1±0·05 | 10/10 |
| | | $5 \times 10^{-5}$ M ATP initially | 4·7±0·2 | 9/10 |
| | | $5 \times 10^{-4}$ M ATP initially | 4·7±0·2 | 10/10 |
| | | $5 \times 10^{-5}$ M Synkavit initially | >26 | 4/10 |
| | | Synkavit + $5 \times 10^{-5}$ M ATP, both initially | 9·6±0·3 | 9/10 |
| | | Synkavit + $5 \times 10^{-4}$ M ATP, both initially | 5·0±0·1 | 10/10 |
| | | Synkavit initially, $5 \times 10^{-4}$ M ATP after 20 min. | 10·0±0·3 | 10/10 |
| D | 30 | .. | 5·0±0·2 | 10/10 |
| | | $1·34 \times 10^{-3}$ M ATP initially | 5·7±0·4 | 10/10 |
| | | $10^{-4}$ M Synkavit initially | >30 | 0/10 |
| | | $10^{-4}$ M Synkavit after 20 min. | 5·3±0·2 | 10/10 |
| | | Synkavit initially, ATP initially | 5·0±0 | 10/10 |
| | | Synkavit initially, ATP after 20 min. | >30 | 0/10 |
| E | 30 | .. | 4·3±0·1 | 20/20 |
| | | $1·1 \times 10^{-4}$ M Synkavit initially | >30 | 0/20 |
| | | Synkavit after 15 min. | 7·8±1·5 | 19/20 |
| | | $8·5 \times 10^{-5}$ M ATP | 4·9±0·3 | 10/10 |
| | | $8·5 \times 10^{-4}$ M ATP | 4·3±0·1 | 9/10 |
| | | Synkavit + $9 \times 10^{-5}$ M ATP | >30 | 0/20 |
| | | Synkavit + $9 \times 10^{-4}$ M ATP after 15 min. | >30 | 0/20 |
| | | $10^{-3}$ M ADP | 4·1±0 | 10/10 |
| | | Synkavit + ADP | 5·0±0 | 10/10 |

All incubations were performed with EAT cells incubated under a gaseous phase of air in Spinner-medium at pH 7·2–7·4 containing 1 g./l. glucose and a cell concentration of $2 \times 10^7$ cells/ml. In A the amount of packed cells injected per mouse was $4·5 \times 10^{-2}$ ml., but in B–E $2·5 \times 10^{-2}$ ml. Other details as Table I.

under an atmosphere of air) with $10^{-4}$ M Synkavit reduced subsequent growth of ascites tumour after inoculation of ascites cells into mice (Tables I and II, Fig. 2). These results may be interpreted in terms of the percentage of cells killed on the basis of Fig. 1, as described previously.

Consequently, a systematic study was made of possible variables which might affect this phenomenon. Fig. 2 shows that the inhibition by Synkavit of tumour growth was dependent on the correct pH of the medium during incubation: in EAT cells, for example, with a 40 minute incubation at pH 7·4 or pH 7·0 (Fig. 2, A) Synkavit inhibited *completely* the growth of tumour; whereas at pH 6·7 the effect was only partial. However, after a 25 minute incubation, complete growth-inhibitory effect was only observed at pH 7·4. Thus, comparison of Fig. 2A and 2B suggests that the extent of the growth-inhibitory effect of Synkavit increased considerably as the incubation time was increased from 25 to 40 minutes. Furthermore Table IID and E, indicates that with EAT cells the inhibitory effect was much smaller when incubation with Synkavit at pH 7·3 was restricted to 10–15 minutes. It may be noted that Fig. 2B shows that the pH-dependence also held true in the case of DV cells, although the effect seemed to be slightly less

FIG. 2.—Dependence of the growth-inhibitory effect of Synkavit on pH and glucose. A and B refer to separate experiments when the times of incubation were 40 and 25 minutes at 37° C. respectively. Closed symbols refer to untreated cells, open symbols to cells treated with $1{\cdot}04 \times 10^{-4}$ M Synkavit. All cells were incubated in Spinner-medium without glucose, except in the case of the experiment denoted by the circles. EAT cells were used in all experiments except those denoted by inverted triangles which involved DV cells. Those tumours which did not grow are counted as growing on the 30th day. ⫱ denotes standard deviation of mean value.

than in the case of EAT cells. The growth-inhibitory effect of Synkavit occurred irrespective of whether glucose was present in the medium or not (Fig. 2A). This fact eliminates two possibilities: (1) that the Synkavit-effect may have depended on the presence of glucose in the medium; and (2) that Synkavit inhibited tumour-growth by virtue of damage to the tumour-cell membrane incurred by adjustment of the pH of the medium (see METHODS, *Incubation conditions*).

The dependence of the effect of Synkavit on aerobic or anaerobic conditions during incubation is illustrated in Table IA and B: in both EAT and DV cells the growth of tumour was retarded only slightly when the cells were incubated in the presence of Synkavit in nitrogen-saturated medium contained in full, sealed bottles. Therefore, the effect of Synkavit on the growth of tumour depends to some extent on the presence of oxygen during incubation (for further comment see DISCUSSION).

The following additional factors which might have affected the growth-inhibitory effect of Synkavit were eliminated: (a) Synkavit was effective when EAT cells were incubated both at concentrations of $10^7$ and $2 \times 10^7$ cells/ml. (Table IC); (b) the effect on EAT cells was not abolished by the presence of about 5% ascitic fluid in the incubation medium (Table ID); and (c) Synkavit inhibited the growth of EAT cells which had been obtained by centrifugation at 50 *g* and 1000 *g* (Table IE).

*Reduction by ATP and ADP of the growth-inhibitory effect of Synkavit*

Table II, A–D, shows that the growth-inhibitory effect of Synkavit on EAT cells may be reduced by simultaneous incubation with 10 × excess, or, less effectively, by equimolar ATP. Consequently, some attempt was made to decide whether or not ATP acted by preventing the incorporation of the active metabolite of Synkavit: Table II, C–E, gives the results of three similar experiments in which excess or equimolar ATP was added either simultaneously or part-way through the incubation of EAT cells with Synkavit. The results given in Table IIC show that 10 × excess ATP added half-way through the 40 minute incubation period with $5 \times 10^{-5}$ M Synkavit reduced the time for tumour growth in much the same way as did equimolar ATP when added initially. However, in the results of the experiments presented in Table IID and E, excess ATP added half-way through the incubation with Synkavit could not reduce the effect of the latter compound; and in the experiment described in Table IIE equimolar ATP added *initially* did not reduce the effect of Synkavit. Therefore, it appears that the magnitude of the Synkavit in this case was greater than that described in Table IIC (although in both cases Synkavit prevented growth of tumour within 30 days).

A different approach to the question of how ATP abolishes the growth-inhibitory effect of Synkavit is to investigate the effect of compounds related to ATP. Thus 10 × excess ADP abolishes the growth-inhibitory effect of Synkavit when the two compounds are incubated simultaneously with the tumour cells (Table IIE).

*Effect of ATP on labelling with TRK* 219

One explanation of the abolition by ATP of the growth-inhibitory effect of Synkavit is that ATP prevented the incorporation of the active metabolite of Synkavit. This possibility may be investigated simply by determining whether or not simultaneous incubation with ATP reduces the incorporation of the tritiated form of Synkavit, TRK 219.

When EAT cells were incubated with TRK 219 for 40 minutes, the presence of 10 × excess ATP throughout the entire incubation time decreased considerably the incorporation of activity (Table III). However, simultaneous incubation with equimolar ATP had only a slight effect (Table III). It is important to note that under these conditions almost all the activity incorporated by the ascites cells was associated with the acid-insoluble fraction.

The results obtained with HEp/2 cells are given in Table IV. In this case, simultaneous incubation with 10 × excess ATP had only a small effect on the incorporation of activity during incubation with TRK 219 for 20 or 40 minutes in either Spinner-medium or bicarbonate-buffered saline. Furthermore, the incorporation of activity was approximately the same when HEp/2 cells were incubated

TABLE III.—*Effect of ATP on Incorporation of TRK* 219 *by EAT Cells*

| | Acid-soluble | Acid-insoluble |
|---|---|---|
| % of total activity associated with fraction | 7;7 | 93;93 |
| % control activity in presence of equimolar ATP | 86;86 | 87;86 |
| % control activity in presence of 10 × excess ATP | 37;39 | 20;24 |

EAT cells ($10^7$/ml.) were incubated with $10^{-4}$ M TRK 219 (62–65 Ci/mM) for 40 minutes in Spinner-medium, pH 7·4, *minus* glucose, in an atmosphere of air. Values given are for duplicate experiments.

TABLE IV.—*Effect of* 10 × *Excess ATP on Incorporation of TRK* 219 *by HEp/2 Cells*

| Incubation time (min.) | Medium | % Total activity acid-insoluble | Effect of ATP % control value | | Ratio uptake in Spinner/saline | |
|---|---|---|---|---|---|---|
| | | | Acid-soluble | Acid-insoluble | Acid-soluble | Acid-insoluble |
| 20 | Spinner | 45+, 55 | 87+, 73 | 63+, 71 | 60+, 94 | 36+, 86 |
| | Saline | 57, 58 | 70, 68 | 72, 73 | 100 | 100 |
| 40 | Spinner | 26 | 78 | 80 | 190, 65=, 175 | 82, 16=, 100 |
| | Saline | 45 | 90 | 110 | 100 | 100 |

Cells (about 6–8 × $10^5$/ml. incubation medium) were incubated with $10^{-4}$ M TRK 219 (59 Ci/mM) at pH 7·4. The pH of the bicarbonate-buffered saline was difficult to control as accurately as that of Spinner-medium.
+: incubated at pH 7·0.
=: cell concentration: 3 × $10^5$ cells/m. incubation medium.

with TRK 219 in Spinner-medium or buffered-saline. Only in one particular experiment, in which the concentration of HEp/2 cells was lower than usual, was the incorporation of activity in Spinner-medium less than that in buffered-saline. Significantly, in the experiments performed with HEp/2 cells, the percentage of the incorporated activity associated with the acid-insoluble fraction was much lower than in the case of EAT cells. This probably reflects the lower concentration of HEp/2 cells in these experiments (about 7 × $10^5$/ml., cf. with EAT cells: $10^7$/ml.).

### DISCUSSION

It is clear from the present results that in the case of both EAT and DV cells incubation *in vitro* with $10^{-4}$ M Synkavit retards their growth if subsequently inoculated into mice, provided that the following conditions are strictly observed:

(a) The pH of the medium must be controlled within the range 7·2–7·4;
(b) For maximum effect, the incubation must be performed aerobically;
(c) For maximum effect, the time of incubation should be not less than 30 minutes.

These conditions are critical and are related to those pertaining to the biochemical effects of Synkavit (Harrison, 1968*a*). Namely, both biochemical and growth-inhibitory effects are pH-dependent in much the same manner; and both occur in the absence of oxygen, although the growth-inhibitory effect is less severe anaerobically. The reason for the latter is not understood, but it is considered unlikely to be due to a better degree of anoxia in the present experiments (for a consideration of this point regarding the biochemical experiments, see Harrison 1968*a*).

The effect of Synkavit on subsequent growth *in vivo* is not dependent on incubation in the presence of glucose *in vitro*. This may be interpreted to mean that the incorporation of the active metabolite of Synkavit into the cell is not glucose-dependent; whereas the biochemical effects of this incorporated metabolite are glucose-dependent. This interpretation is supported by the finding by Dr. Valerie Fisher (personal communication) that the dephosphorylation of Synkavit *in vitro* is *not* glucose-dependent. Since the primary biochemical effect of Synkavit was considered to be the reduction of the synthesis and cellular content of ATP, it may be reasonably argued that once some metabolite of Synkavit has been incorporated into the cells *in vitro*, it is able to uncouple the energy-yielding processes whereby the glucose supply *in vivo* is utilised for tumour growth.

It is pertinent to consider at this point the recent report by DiVita and Marrian (1969). The experimental conditions used by these workers differed from those used by the present author in only three respects: the lower cell concentration during incubation, the presence of ascitic fluid in the incubation medium, and the speed of centrifugation of the cells. Each of these differences in technique has been shown in the present work not to reduce the growth-inhibitory effect of Synkavit. Nevertheless, DiVita and Marrian, using groups of 30 mice, were *not* able to detect any effect of Synkavit on the growth of DV cells when incubated under *anaerobic* conditions for 30 minutes and at concentrations of $10^{-4}$ M or $10^{-2}$ M in Spinner-medium containing 1 g./litre glucose or physiological saline at an initial *pH of* 7·0–7·5. Thus, on the basis of the present work, it is evident that DiVita and Marrian did not realise, in their choice of anaerobic conditions and wider pH range, the optimum conditions for the growth-inhibitory effect of Synkavit. Moreover, care must be taken in judging the significance of experiments performed with high concentrations of Synkavit: Dendy (1969) has evidence to show that in those types of cells which show a selective uptake of the drug (to which both DV and EAT cells appear to belong—see Dendy, 1970), at concentrations higher than $3{\cdot}15 \times 10^{-4}$ M the uptake of a tritiated derivative of Synkavit (TRK 219) falls in absolute terms as the molarity is increased. In fact, Dr. D. H. Marrian (personal communication) has recently observed a large inhibition of tumour growth by $10^{-4}$ M Synkavit when EAT cells were incubated in air for 40 minutes in Spinner-medium *minus* glucose, and maintained at pH 7·3–7·4.

Finally in this context, it may be noted that Dendy (1970) has evidence that those types of cells which show a selective uptake of tritiated Synkavit are selectively killed, as judged by cloning experiments.

*Effect of ATP*

The simplest interpretation of the fact that ATP may reduce the growth-inhibitory effect of Synkavit would be that it reduced the incorporation of the active metabolite of Synkavit. This may be envisaged as competition between ATP and Synkavit at the enzymic dephosphorylation sites which are thought to be involved in the conversion of Synkavit to its active metabolites. For the sake of clarity, the experiments concerning 10 × excess or equimolar ATP will be discussed separately.

(i) 10 × *excess ATP*.—From the experiments described previously, it is clear that incubation of EAT cells with TRK 219 plus 10 × excess ATP reduces the incorporation of activity by about 80%. This may be compared with a complete reduction in the growth-inhibitory effect of Synkavit when incubated simultaneously with 10 × excess ATP. Thus, the simple competition hypothesis would explain these results adequately. The results obtained when 10 × excess ATP was added half-way through the incubation with Synkavit do not permit definite conclusions to be drawn. Nevertheless, these results are not inconsistent with the competition hypothesis. Moreover, the experiment involving 10 × excess ADP would be readily explained if it is assumed that ADP could saturate the dephosphorylation sites in a similar manner to ATP.

The results presented for HEp/2 cells are not relevant to the growth-inhibitory effect of Synkavit. Nevertheless, they do show clearly that 10 × excess ATP

inhibits only slightly the incorporation of activity from TRK 219 by these cells, either when incubated in Spinner-medium or bicarbonate- buffered saline. Thus, simple competition between ATP and Synkavit is not necessarily observed in cells which show a selective uptake of TRK 219 (Dendy, 1970).

(ii) *Equimolar ATP*.—Simultaneous incubation with equimolar ATP was shown to reduce the incorporation of activity from TRK 219 into EAT cells by 14%. The corresponding reduction in the growth-inhibitory effect of Synkavit was more variable. However, on the basis of Fig. 1, the reduction can be estimated to be about 15–40%, in terms of the number of cells killed or retarded in growth. Thus, in view of the errors involved in these comparisons, these results do not disprove the simple competition hypothesis.

However, certain other observations (Fisher, personal communication) must be considered in this context. Her studies of the rates of dephosphorylation of mixtures of ATP and Synkavit by EAT cells suspended in phosphate-free medium (containing the same metal ions as Spinner-medium, but buffered with imidazole) led her to believe that ATP stimulated the dephosphorylation of Synkavit. The same author showed that imidazole did not affect the dephosphorylation of Synkavit, and deduced from the different pH-dependences and responses to inhibitors that the enzymic sites for dephosphorylation of Synkavit and ATP were probably not identical. Consideration of these results in relation to those described in the present studies may suggest that in EAT cells inorganic phosphate may play some intermediate role in controlling competition between dephosphorylation of Synkavit and ATP, possibly at different enzymatic sites. Unfortunately, the results obtained by Dendy (1970) on the uptake of TRK 219 in Spinner-medium and phosphate-free medium do not permit any conclusion on this point.

The author wishes to thank Professor J. S. Mitchell, F.R.S., for his interest and encouragement; Mrs. M. A. Williams for helpful technical assistance; Mr. E. A. King who has performed the inoculations of the mice; and Dr. D. H. Marrian for reading the manuscript. The work described in this paper was initiated during the tenure of a Medical Research Council Scholarship for Training in Research Methods and completed whilst the author held a Beit Memorial Research Fellowship and a Research Fellowship at Trinity Hall, Cambridge.

### REFERENCES

DENDY, P. P.—(1969) *Acta radiol. Ther. Phys. Biol.*, **8**, 513.—(1970) *Br. J. Cancer*, **24**, 817.

DIVITA, G. AND MARRIAN, D. H.—(1969) Proc. 2nd int. Symp. on Radiosensitizing and Radioprotecting Drugs. (Rome) p. 225.

GILL, D. M.—(1967) *Int. J. appl. Radiat. Isotopes.*, **18**, 393.

HARRISON, P. R.—(1968*a*) *Br. J. Cancer*, **22**, 274.—(1968*b*) Ph.D. dissertation, Cambridge University.

MITCHELL, J. S.—(1951) 'Studies in Radiotherapeutics'. Oxford (Blackwells).

ROSSI, M. AND DIVITA, G.—(1960) *Boll. Soc. ital. Biol. sper.*, **36**, 1340.

WARBURG, O.—(1956) *Science, N.Y.*, **123**, 309.